# Socioeconomic Disparities in Social Distancing During the COVID-19 Pandemic in the United States: Observational Study

**DOI:** 10.2196/24591

**Published:** 2021-01-22

**Authors:** Romain Garnier, Jan R Benetka, John Kraemer, Shweta Bansal

**Affiliations:** 1 Department of Biology Georgetown University Washington, DC United States; 2 Unacast Oslo Norway; 3 Department of Health Systems Administration Georgetown University Washington, DC United States

**Keywords:** COVID-19, SARS-CoV-2, disease ecology, nonpharmaceutical interventions, mobility data, economic, disparity, social distancing, equity, access, socioeconomic, infectious disease, mobility

## Abstract

**Background:**

Eliminating disparities in the burden of COVID-19 requires equitable access to control measures across socio-economic groups. Limited research on socio-economic differences in mobility hampers our ability to understand whether inequalities in social distancing are occurring during the SARS-CoV-2 pandemic.

**Objective:**

We aimed to assess how mobility patterns have varied across the United States during the COVID-19 pandemic and to identify associations with socioeconomic factors of populations.

**Methods:**

We used anonymized mobility data from tens of millions of devices to measure the speed and depth of social distancing at the county level in the United States between February and May 2020, the period during which social distancing was widespread in this country. Using linear mixed models, we assessed the associations between social distancing and socioeconomic variables, including the proportion of people in the population below the poverty level, the proportion of Black people, the proportion of essential workers, and the population density.

**Results:**

We found that the speed, depth, and duration of social distancing in the United States are heterogeneous. We particularly show that social distancing is slower and less intense in counties with higher proportions of people below the poverty level and essential workers; in contrast, we show that social distancing is intensely adopted in counties with higher population densities and larger Black populations.

**Conclusions:**

Socioeconomic inequalities appear to be associated with the levels of adoption of social distancing, potentially resulting in wide-ranging differences in the impact of the COVID-19 pandemic in communities across the United States. These inequalities are likely to amplify existing health disparities and must be addressed to ensure the success of ongoing pandemic mitigation efforts.

## Introduction

Treatment options and vaccines are being developed to address the COVID-19 pandemic [1]. However, while the ability to detect new infections remains limited by testing capacity and the lack of nationwide syndromic surveillance capabilities [2], nonpharmaceutical interventions represent the only immediate tools public health agencies can use to limit the size and spatial scale of the outbreak [3]. In the United States, state and local governments are primarily responsible for measures such as school or business closures [4]. Historically, similar measures have been used to respond to pandemics, including during plague outbreaks in the Middle Ages [5] and during the 1918 Spanish influenza pandemic [6]. Data collected during the early part of the ongoing COVID-19 pandemic, in particular on the dynamics of the outbreak in China, indicate that nonpharmaceutical interventions can be successful in limiting the size of COVID-19 outbreaks [7] and in delaying large-scale spread [3].

However, social distancing may be adopted differently across communities, especially in the United States, where workers in sectors such as transportation and food retail receive lower wages and represent a larger fraction of workers deemed essential than those in other sectors of the workforce [8,9]. Assessing this differential impact requires the use of fine-scale mobility data, a stream of information that has proven useful in the early assessment of social distancing measures in the United States [10], Italy [11], and France [12]. Digital technologies have taken center stage in the response to COVID-19 [13], and mobility data in particular have enabled assessment of the responses to nonpharmaceutical interventions [14,15]. Previous studies report large-scale reductions in movement, with numbers quickly reaching values typically observed during holiday periods [10]. Furthermore, in the United States, changes in mobility were associated with reductions in COVID-19 cases [16,17] and in the reproduction number of the disease [18], and mobility data also revealed that these changes were largely already underway when state or county stay-at-home orders were issued [16,19,20]. Previous studies using mobility data have also suggested potential inequalities in the ability to practice social distancing based on income [21], race and education [20], or the availability of health care providers [22]. However, most of these studies consider these determinants in isolation and do not allow to disentangle the potential additive effects of the socio-economic make-up of counties on the ability of their populations to practice social distancing. These studies have also largely focused on understanding how social distancing was influenced by local or regional decisions and whether socioeconomic factors changed the responses to state and local interventions.

Here, we focus on an ecological understanding of how mobility varies with socioeconomic characteristics rather than assessing what drives these changes. As outlined above, multiple studies have aimed to understand the causes of mobility behavior changes. However, we seek to understand how the patterns of mobility vary across socioeconomic characteristics (regardless of the cause) during different stages of the pandemic response. In particular, we ask how quickly, how deeply, and for how long mobility changes occurred in locations according to their socioeconomic characteristics. Our approach does not seek to differentiate between spontaneous changes in mobility, such as in response to news coverage, or changes in response to state- or county-mandated orders. Rather, we focus on the resulting changes in mobility and how these differ by location and socioeconomic status.

## Methods

To measure mobility, we obtained daily county-specific mobility data for the United States from February 24 to May 14, 2020—the period during which most of the United States was simultaneously engaged in social distancing—through a partnership with Unacast [23]. The data set is based on the GPS location data collected from applications installed on tens of millions of devices, and it complies with the General Data Protection Regulation and the California Consumer Privacy Act [24]. The data set was shown to be representative by geographical location, income level, sex, and age in an analysis conducted by Unacast [25]. The fraction of all devices observed varies by location and time, and this has been captured in our analysis (details below).

As outcome measures, we considered data on the changes in three measures of mobility provided by Unacast: daily distance traveled (hereafter, “distance traveled”), rate of visitation to nonessential places (hereafter, “visitation rate”), and rate of encounters between devices within a 50-meter radius within an hour (hereafter, “encounter rate”). Distance traveled reflects the average distance between the home locations of users and locations visited in 1 day. Visitation rate reflects the number of visits to nonessential locations; the definition of nonessential venues is based on state-specific guidelines and policies and includes all locations other than those deemed essential (eg, food stores, pet stores, and pharmacies; more information can be found on Unacast’s website [26]). Encounter rate measures the likelihood of proximity between any two users within 50 meters over a one hour period. Each change is calculated relative to a county-specific baseline calculated from values obtained during a period of several weeks prior to the onset of major COVID-19–related changes in mobility in the United States (February 10 to March 4, 2020 for distance traveled and visitation rate; February 24 to March 4, 2020 for encounter rate). The resulting data set covers 3054 counties for the distance traveled and encounter rate and 2067 counties for the visitation rate. The county-level data on each measure described above can be accessed by contacting Unacast [23], and our model-processed data and the code used for the statistical analysis are available on GitHub [27].

**Figure 1:**
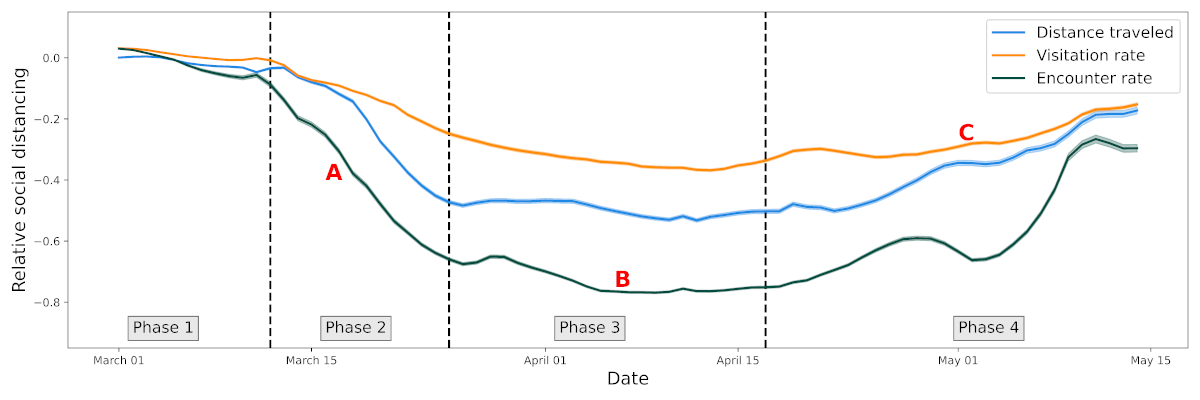
Time series of three mobility measures aggregated at the country level. We show a 7-day rolling mean of distance traveled (blue), visitation rate (orange), and encounters rate (green), with the solid line representing the mean and the shaded area two standard errors of the mean. The 4 phases in the mobility time series are delimited by dashed vertical lines in the figure and were generated from the model fit in each county independently. These phases allow us to calculate three summary measures for each time series: the slope of decline in phase 2 (A), the mean level of mobility during social distancing (B), and the slope of increase in mobility during exit from social distancing (C).

To summarize the mobility time series, using the `fbprophet` package [28], we first fit a nonlinear model to the county-level social distancing time series, including a weekly trend to account for workweek variation. This package fits a piecewise regression while allowing setting the number of potential changepoints, and makes it possible to assess where breaks in the trend occur over the course of the social distancing time series. Most counties follow a dynamic similar to that of the mobility measures aggregated at the country level (Figure 1). In short, the underlying trend can be separated into four phases (ie, four breaks in the trend): phase 1, the baseline period; phase 2, the period of entry into social distancing, measured by the rate of mobility decrease; phase 3, the social distancing period, described by a sustained reduction in mobility outcomes; and phase 4, the period of exit from the social distancing phase, measured by the rate of mobility increase after sustained social distancing. This dynamic is specific to 2020; these four phases are not evident in 2019 (Figure S1 in Multimedia Appendix 1). From these model fits, we extracted several values that enabled us to characterize the changes in mobility during entry into and exit from the social distancing phase, as well as the mobility level during the period of sustained mobility reduction (see Figure 1 for details). Counties with no detectable increase in mobility after sustained social distancing (ie, where the slope of the trend at the end of the time series remains nonpositive) were not included in the phase 4 analysis. Each county time series was thus summarized by 3 values (or 2 values for the counties with no detectable phase 4 increase), which were used in the statistical analysis. We do not distinguish whether these changes are spontaneous in response to the COVID-19 pandemic or occur in response to public health policies; we only define the phases based on changes in mobility rates. That is, we do not seek to explain why the mobility changed but rather how it changed.

Our exposures of interest relate to the socioeconomic composition of each area: racial composition, population density, proportion living below the poverty level, and proportion of the workforce in industries designated as essential. Thus, our exposures of interest are area-level features, not individual features. We obtained information on racial composition and population density from the 2018 American Community Survey [29] and on the proportion of people below the poverty level from the Small Area Income and Poverty Estimate program [30]. We estimated the proportion of workers in industries designated as essential [31] from the Quarterly Census on Employment and Wages for the fourth quarter of 2019 [32].

We also adjusted for the fractions of devices observed in each county because sampling of mobile devices tends to vary geographically and over time.

We ran linear mixed models to analyze the associations between the social distancing summary values and the socioeconomic variables with state as a random effect using the standard 0.05 significance threshold. In the main analysis, we investigated independent associations with each covariate, and the resulting linear mixed model is of the form:

*Y* = *X_i_β_i_* + *Z_μ_* + *ε*

In our case, the response variable *Y* is one of the social distancing summary measures (the slope in phase 2, mean value in phase 3, or slope in phase 4). The socioeconomic predictors are included as the fixed effects *X_i_* and the state as the random effect *Z*. The *ε* term captures the residuals. We ran independent models for each of the mobility measures (distance traveled, visitation rate, and encounter rate) and for each of the social distancing summary measures. We also performed a post hoc secondary analysis in which interaction terms between covariates were added to elucidate findings from the main analysis. Finally, as a sensitivity analysis, we fit an alternative linear model with state as a fixed effect rather than a random effect to adjust for any additional unmeasured state-level features. All analyses were conducted in Python 3.6.

The research presented in this paper was approved by the Georgetown-Medstar Institutional Review Board (study id STUDY00003041).

## Results

**Figure 2:**
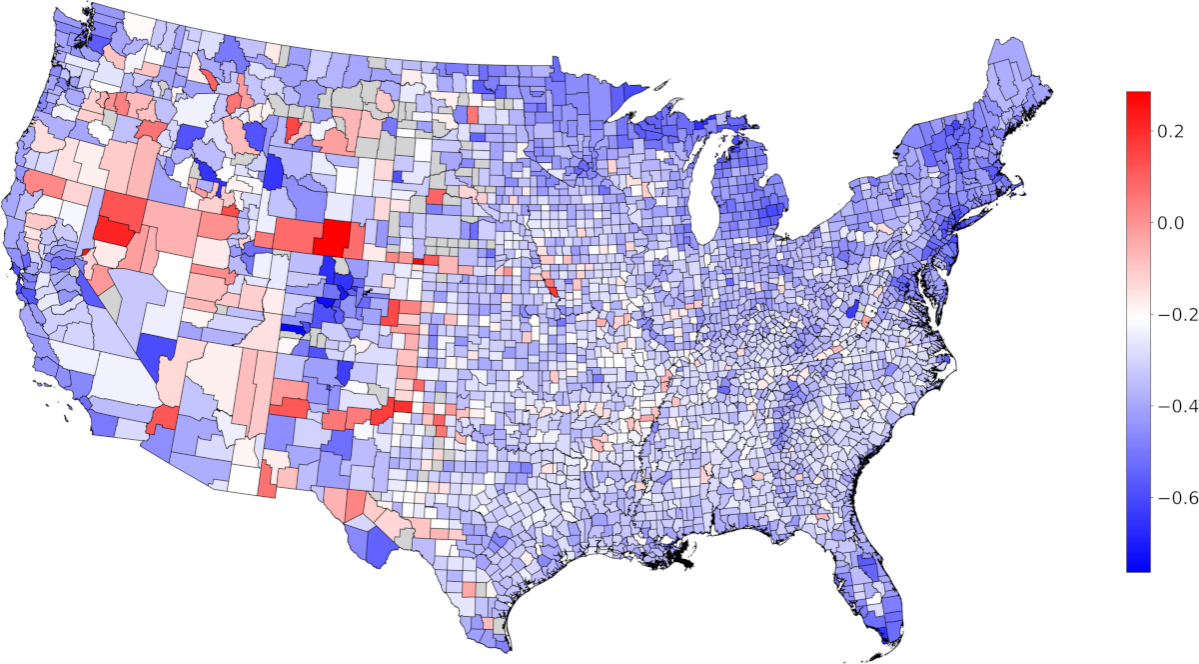
Heterogeneity in mobility during social distancing. The map shows the average mobility during social distancing due to COVID-19 at the county level in the continental United States relative to the pre–COVID-19 baseline. A positive value indicates an increase in distance traveled, and a negative value indicates a decrease in distance traveled. Counties for which data are not available are shown in grey. The color map is centered at the 90% percentile of the decrease in mobility.

**Figure 3:**
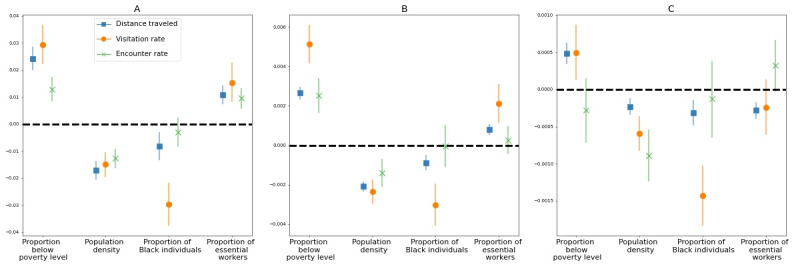
Regression coefficients of distance traveled, visitation rate, and encounter rate for the 4 socioeconomic factors associated with social distancing: (A) mobility during social distancing (phase 3); (B) decline in mobility during entry to the social distancing phase (phase 2); and (C) resurgence in mobility during exit from the social distancing phase (phase 4). The marker denotes the mean coefficient, and the error bars show the 95% confidence interval. A positive association (above the dashed line) indicates that an increase in a given factor leads to a weaker implementation of social distancing. A negative association (below the dashed line) indicates that an increase in the given factor is associated with a stronger implementation of social distancing measures.

Social distancing is heterogeneous at the county level (Figure 2). In counties with a higher proportion of people in poverty, social distancing was weaker: mobility was less restricted during the period of sustained mobility reduction, and the change occurred more slowly during the period of entry into social distancing for all three measures of mobility. Additionally, the resurgence in mobility was faster during the period of exit from sustained reductions in mobility for 2 of 3 measures of mobility (Figure 3). Counties with higher proportions of essential workers saw weaker social distancing adoption for all mobility markers during the period of sustained mobility reduction and a slower entry into social distancing based on two mobility markers. The rate of exit from the social distancing phase is less predicted by the proportion of essential workers. Contrastingly, in counties with a larger proportion of Black individuals or a higher population density, social distancing is stronger, with a faster entry into social distancing, lower mobility levels during the period of sustained mobility reduction, and a slower resurgence during the period of exit from the social distancing phase (Figure 3). The results were not significant for the encounter rate for the proportion of Black individuals in the population. Full statistical details are provided in Table S1 (Multimedia Appendix 1).

When interaction effects are added to the model of distance traveled in the period of entry into mobility reductions, all variables follow the same qualitative and quantitative patterns (Table S2, Multimedia Appendix 1). We found a significant positive interaction between the proportion of essential workers and the Black population, and we found a negative interaction between essential workers and low-income workers. The interaction between the proportions of Black people and low-income workers is not significantly associated with mobility.

Substituting the random effect state with a fixed effect yielded very similar results. There were only three differences of note: the association between the mobility resurgence measure as the visitation rate in the period of exit from the social distancing phase and the proportion of low-income workers became nonsignificant, while the associations between the resurgence in encounter rate in the period of exit from the social distancing phase and the proportions of essential and of low-wage workers became significant.

## Discussion

The COVID-19 pandemic has highlighted significant health disparities in the United States, similar to the health inequities driven by income inequality and racial injustice that previously existed in the country [33]. Understanding the role of behavioral interventions in driving variations in the COVID-19 burden is crucial to our current and future outbreak response. Our study shows that changes in interaction in response to the pandemic are geographically heterogeneous and are associated with county-level socioeconomic factors. This is true for both the level of mobility restriction implemented during the social distancing phase and for the rate at which populations enter (“response engagement“) and exit (“response fatigue”) the social distancing phase.

Our analysis reveals that the occupational composition of the counties is associated with how deeply and for how long social distancing is maintained. Populations including more essential workers, who maintained food services, public transportation, and health care services during the pandemic [8], understandably participate less in social distancing and thus experience greater risk. Additionally, lower-income populations participate less in social distancing, likely in part because low-wage workers may have less access to job protections or paid leave. Our results provide further nuance to the analysis by Lou et al [9], who found that lower-wage workers were unable to reduce their work trips, in large part because businesses classified as essential tended to pay lower wages. Our results may help explain why lower-income counties have suffered a disproportionately high death burden from COVID-19 [17]; however, these results also need to be taken in light of the more general role that low income plays in negative health outcomes [21]. Reduced access to employer-sponsored health care [34] could further limit testing and treatment-seeking behavior and potentially worsen outbreaks in these communities. In rural communities (those with low population density in our study), the need to travel farther to access essential supplies and services such as food or health care [35] may also limit social distancing and would further confirm the existing disparity whereby rural counties suffer from poorer health outcomes than their more urban counterparts [36].

Importantly, we also found that counties with larger Black populations showed stronger adherence to social distancing measures during all phases, after controlling for the effects of income, occupation, and density. Our finding is supported by more local observations of differences between predominantly Black and White neighborhoods, such as Detroit [37]. We also found that social distancing remains more limited in populations that combine high proportions of Black individuals with high proportions of essential workers, possibly because minorities may be overrepresented in certain essential occupations [8]. Despite stronger distancing, there is growing evidence that African American communities experience higher rates of infection and death from COVID-19 [17,38,39]. We advocate for additional work on the structural racism that is at the root of these health disparities [40] and on the role of privilege in the differential burdens imposed by COVID-19 on a variety of communities [22,41].

There is a risk of the ecological fallacy if our results are interpreted as applying to individuals with the attributes we investigated rather than the share of attributes in communities. Survey and qualitative studies would help explain how individual, community, and public policy-level factors explain these associations.

Without large-scale test-trace-isolate programs or other interventions, intermittent social distancing will continue to be needed to contain cases and minimize the strain on health systems [42]. Technological solutions are being suggested and to an extent implemented [13-15]; however, these solutions are not without their limitations. The large-scale use of mobility data and other digital technologies (eg, for contact tracing) has opened up a debate on the responsible use of these emerging data streams [13,43], for instance to ensure that privacy concerns are properly assessed and addressed. These technologies would also likely be most effective with the implementation of a spatially and socially homogeneous testing strategy. Similarly, the long-term success and equity of a mitigation strategy hinges on paying more attention to the geographic heterogeneity in outbreak mitigation and focusing on the role of social and employment policies that affect the ability of individuals to engage in behavioral interventions.

## Appendix

Multimedia Appendix 1 Supplemental material.
